# Acceptability of short message service (SMS) as a tool for malaria treatment adherence in the Brazilian Amazon: a qualitative study

**DOI:** 10.1590/0037-8682-0616-2022

**Published:** 2023-05-22

**Authors:** Sheila Rodovalho, Ádila Liliane Barros Dias, Maria Paz Ade, Diego Macias Saint-Gerons, Jose Luis Castro, Andrea Beratarrechea, Felipe Leão Gomes Murta, Alicia Cacau Patrine dos Santos, Leonardo Lincoln Gomes Marques, Vanderson Souza Sampaio, Djane Clarys Baia-da-Silva, Wuelton Marcelo Monteiro

**Affiliations:** 1 Universidade do Estado do Amazonas, Programa de Pós-graduação em Medicina Tropical, Manaus, AM, Brasil.; 2 Organização Pan-Americana da Saúde, Organização Mundial da Saúde, Departamento de Doenças Transmissíveis e Determinantes Ambientais da Saúde, Brasília, DF, Brasil.; 3 Fundação de Medicina Tropical Dr. Heitor Vieira Dourado, Manaus, AM, Brasil.; 4 Pan American Health Organization, Department of Communicable Diseases and Environmental Determinants of Health, Washington, USA.; 5 Pan American Health Organization, Department of Health Systems and Services, Unit of Medicines and Health Technologies, Washington, USA.; 6 Institute of Clinical Effectiveness and Health Policy, Buenos Aires, Argentina.; 7 Fundação de Vigilância em Saúde do Amazonas, Manaus, AM, Brasil.; 8 Fundação Oswaldo Cruz, Instituto de Pesquisa Leônidas & Maria Deane, Manaus, AM, Brasil.

**Keywords:** Malaria, SMS, Medication adherence, Malaria treatment

## Abstract

**Background:**

Malaria is one of the leading causes of morbidity worldwide, and patient adherence to prescribed antimalarials is essential for effective treatment.

**Methods:**

This cross-sectional study, with in-depth telephone interviews, analyzed participants’ perceptions of short message service (SMS) in adherence to treatment.

**Results:**

Five thematic categories emerged: decreased forgetfulness, the novelty of the tool, easy-to-understand language, the impact of SMS messages during treatment, and suggestions for improvement and complaints.

**Conclusions:**

SMS could assist patients in adhering to prescribed antimalarials.

Malaria is a leading cause of morbidity worldwide, with billions of individuals living in areas of high transmission^1^. *Plasmodium vivax* is the main causative agent of malaria in Brazil, with high frequency in the Brazilian Amazon, where recurrence is responsible for a high disease burden and increased transmission^2^. The Brazilian Ministry of Health recommends a three-day chloroquine and seven-day primaquine regimen to combat uncomplicated vivax malaria^3^. However, this regimen may limit the success of antimalarial treatment owing to low patient adherence^4^. Studies have attempted to evaluate improved strategies to increase adherence, including direct supervision, community education, treatment instructions, and short message service (SMS) text messages^4^ ^,^ ^5^. 

The telecommunications sector in Brazil is growing annually, and mobile telephony has reached a total of 255.7 million cell phones, primarily with 4G technology (77.5%), followed by 3G (11.1%) and 2G (10.8%)^6^.

The availability of 4G networks in Brazil is growing rapidly, with operators expanding their long-term evolution (LTE) reach and investing in a new spectrum^7^. Adopting new 4G frequencies in the 700 MHz band could profoundly impact 4G availability scores, both at the national level and in cities where these 700 MHz networks have recently been activated. Given that 700 MHz operates at a relatively low frequency, signals propagate further, extending coverage in rural areas and improving indoor penetration in urban areas. 

In Manaus, mobile coverage is good when compared with that in other parts of the Brazilian Amazon, which can be attributed to well-developed telecommunications infrastructure and support from major mobile phone operators. Remote, hard-to-reach areas, including the Amazon rainforest and riverside communities, may encounter limited mobile network coverage^6^.

Saint-Gerons et al.^4^ have evaluated a multicomponent strategy to improve adherence to malaria treatment in Manaus, Amazonas, using a Smart Security Surveillance (3S) approach. Messages varied in number from three to seven, including welcome messages, adherence reminders, positive reminders, safety reminders, and an end message. Participants could receive SMS messages free of charge. A high adherence rate (93.3%) was noted on providing an educational package and SMS messages; however, perceptions of SMS intervention were not evaluated. 

Herein, we qualitatively evaluated the perception of SMS in terms of adherence to *P. vivax* treatment among participants from the Saint-Gerons et al.^4^ cohort, who were randomly selected and invited to participate in telephone depth interviews (TDIs). The number of participants with TDIs was determined based on the principle of theoretical saturation. Interviews were conducted using a semi-structured script with 17 previously validated open-ended questions (Table 1). The number of TDIs was determined based on the theoretical saturation^8^.

**TABLE 1:** Script of the interview applied to patients diagnosed with malaria and who received free SMS text messages during the treatment period.

Questions / Theme	Objective
1. What is your name?	Getting to know the participant and his/her personal experience with the tool. Bring the interviewer and the participant closer
2. Did you receive SMS messages to remind you about malaria treatment?	
3. How was your experience with these messages?	
4. Have you ever seen anything like it before? (If yes, do you have an example?)	
5. Did the SMS use easy-to-understand language?	
6. Did you have any difficulty understanding anything? (If yes, what?)	
7. How did you feel about this approach?	Assess the acceptability of the tool
8. Did you like receiving messages? Why?	
9. Was there anything you did not like? (If yes, what?)	
10. Is there anything you identified as negative in receiving these messages?	
11. Do you think it is useful to receive SMS messages, or do you prefer to remember to take the treatment yourself?	
12. Do you believe that the use of this tool will reduce forgetfulness when taking malaria treatment?	
13. Do you think the tool could be improved? (If so, how?)	
14. Did you stop taking your malaria treatment at any time? If yes, why?	Identify the reason for non-compliance
15. Did you receive the messages on the right days, or did they fail at some point?	
16. Did you forget to take your treatment? What did you do?	
17. Did you have a problem with your treatment?	

To ensure superior reliability of data collection, a previous validation of the script was conducted with a smaller sample of people who received SMS messages. The script was developed by a team experienced in qualitative research. TDIs were performed by two trained researchers (APCS and LLGM) between March 2021 and June 2021. The interviews lasted for approximately 20 min. After data collection, TDIs were transcribed and analyzed using MAXQDA21. Qualitative analysis was performed via an analysis of the thematic framework, and analytical categories were created after reading interview transcripts^9^.

All study participants provided informed consent to participate and receive SMS text messages and follow-up phone surveys. Ethical approval was obtained from the Ethical Review Committee on Research (PAHOERC) and the Ethics Committee at the Fundação de Medicina Tropical Doutor Heitor Vieira Dourado (CAAE:18305819.4.0000.0005). 

Herein, 14 participants were recruited to conduct TDIs, and the analysis of their interviews revealed five distinct thematic categories: decreased forgetfulness, novelty of the tool, easy-to-understand language, impact of SMS messages during treatment, and suggestions for improvements and complaints. Among the participants, nine were male and five were female, with an average age of 37 years. Notably, 12 participants had completed high school education, while only two males had completed elementary school.

Most participants believed that SMS messages are important to ensure comprehensive treatment adherence, as they help reduce forgetfulness when taking medications during daily tasks and concerns. 

*“I think it will help a lot. It will not be 100% effective, but it will definitely help.”* (Participant 07)

*“It is useful to receive messages because, at least, we know that we have to take the medicine correctly, because when we are alone, we usually forget. Then, when the messages arrive, no, the messages would reduce forgetfulness.”* (Participant 14).

*“Those who are sick want to take the medicine. The problem is that they forget, as they have a child to take care of; hence, I think this tool is really good.”* (Participant 11)

For some participants, SMS messages as a tool to ensure treatment adherence was valuable, with no previous exposure to this tool. SMS messages were considered an additional patient care strategy desired by most interviewed participants. 

*“I found it interesting because they had not done this in any basic health unit or for any other disease (...) I liked it.”* (Participant 05)

*“Usually, they just give the medicine, and that is it. This was the first time.”* (Participant 14).

The interviewees stated that SMS messages employed adequate, clear, and objective language, allowing easy understanding; however, some participants needed assistance in reading the SMS messages.

*“It was not a long SMS message. I had no difficulty reading and understanding it.”* (Participant 09)

*“It was smooth and very easy. It told me the time to take the medicine. I liked it. It was quite interesting.”* (Participant 03)

*“Sometimes I did not even open the SMS messages, as I did not understand, but when my daughter explained them, I understood.”* (Participant 08)

According to participants undergoing treatment, sending SMS messages was helpful, reminding them to take medications and complete their treatment on time, demonstrating the usefulness of SMS messages as a reminder tool.

*“Yeah, I was taking it when they sent the messages.”* (Participant 01)

*“I thought it was good. At least I remembered; I did not forget.”* (Participant 02)

*“I thought it was good, as I said, I thought it was good because it was something to help us take the medication, because, in fact, when I got sick, I took it, but I forgot the medication, then malaria came back. This was before I got the messages. Then, when I went back, they started texting me.”* (Participant 04)

Although participants found SMS messages important for ensuring treatment adherence, they did provide some suggestions. Their preferences for WhatsApp (a cross-platform instant messaging and voice calling application for smartphones) and calls were identified.

*“They could send messages through WhatsApp. It is better. People read them more often.* SMS *messages only come from debt collectors, and we hardly ever look at them anymore. I, for example, hardly read them. I check WhatsApp* messages *more frequently.”* (Participant 02)*.*

*“For me, it is great, but it would be important to be via a call. Numerous people forget to check their SMS messages. The phone calls we see. People usually have no internet or WhatsApp, so a phone call is better.”* (Participant 14).

Telemedicine has also been suggested to improve patient adherence. Adding further information in SMS messages, for example, regarding what to do when you are sick and/or malaria treatment, was suggested.

*“In SMS messages, you can put what you can or cannot do, what you can take, what can improve, what can get worse”* (Participant 15)*.*

*“There should be a phone number so we can say ‘I’m sick, I have malaria’. (...) There should be ease of care, just like they are doing with COVID. That would help a lot.”* (Participant 09)

Some participants reported inconsistencies in timing and days between medication administration and the arrival of SMS messages.

*“The medication was to be taken in the morning, and the SMS message arrived in the afternoon. The timing was not right*.” (Participant 07)

Interviewees had a good perception of SMS messages as a tool to assist in adherence to prescribed antimalarial drugs; however, they also suggested new tools. Interviewees evidenced the practical usefulness of SMS messages in reducing forgetfulness when taking medications. Forgetfulness is an important barrier to precise drug adherence; therefore, technologies such as SMS messages could be used for frequent reminders^10^. Mobile applications and SMS text messages have been identified as effective mobile health interventions to aid public health services in improving adherence^11^. mHealth tools must be routinely evaluated to ensure their effectiveness in various populations, contexts, and diseases.

The participants found that SMS messages were presented in an easy-to-understand language. However, some participants could not read or understand or found the language used incomprehensible, which may be directly related to low levels of education or illiteracy, common in malaria-endemic areas, demonstrating an important limitation of this technique (SMS). It is important to highlight that health communication strongly correlates with improved medication adherence; therefore, training physicians, other health professionals, and mHealth tool programmers to improve and simplify language can facilitate and increase adherence to health practices, as well as improve health and drug treatment^12^.

The participants were grateful for the additional follow-up and care via text messages. The care and concern offered by SMS messages to participants in relation to their clinical condition is a positive perception of SMS messages in improving medication adherence, which has been established in other qualitative studies^10^. Although several participants found SMS messages to be an innovative healthcare and assistance treatment tool, some suggested using WhatsApp communication as an alternative, given that text messages were deemed irrelevant owing to preexisting associations in their daily lives. Access to reliable Internet and cellular services is crucial for the success of mHealth interventions, especially in areas with a high incidence of diseases such as malaria. The Brazilian government's *Amazônia Conectada Project* (PAC) aims to extend the telecommunications infrastructure network by installing a fiber-optic cable network in the remote Amazon region. Implementing this network will provide access to services such as cell phone coverage, internet, and telemedicine, ultimately improving healthcare outcomes in the region^13^. However, in areas lacking cellular network access, alternative technologies such as satellite communication may be necessary to enable the use of mHealth tools, such as satellite internet^8^ ^,^ ^9^. Implementing traditional healthcare delivery methods may be challenging in such areas, making mHealth a valuable alternative for improving healthcare outcomes^14^. Healthcare providers can use SMS messaging to communicate with patients and provide reminders regarding medication adherence or appointments. Moreover, SMS messaging can be used to provide health education and information to patients regarding malaria and other neglected diseases. The mHealth strategy could close the gaps between patients and health systems. However, access to these technologies is limited in certain communities that live in areas with a high malaria burden and restricted access to the internet or cellular networks, such as riverine communities, indigenous populations, and quilombolas. The findings of the present study should be interpreted with caution, given the considerable limitation of the small sample size and non-inclusion of participants from urban and peri-urban areas from the Brazilian capital, where mobile phone use is routine. 

In conclusion, interviewees demonstrated a positive perception of SMS messages as a tool for assisting in adhering to prescribed antimalarials. 
